# Determinants of Male Partner Involvement during Antenatal Care among Pregnant Women in Gedeo Zone, South Ethiopia: A Case-Control Study

**DOI:** 10.5334/aogh.3003

**Published:** 2021-02-17

**Authors:** Zerihun Berhanu Mamo, Selamawit Semagn Kebede, Selamawit Dires Agidew, Moges Mareg Belay

**Affiliations:** 1 Department of Reproductive Health, School of Public Health, College of Health Sciences and Medicine, Dilla University, Dilla, Ethiopia

## Abstract

**Background:**

Male involvement during antenatal care is an influential strategy for improving maternal health service utilization, especially institutional delivery. In Ethiopia, only one-fifth of pregnant women were accompanied to antenatal care. It is among those neglected issues, as it is not well studied, specifically determinant factors of male involvement during antenatal care are not known.

**Objective:**

This study aimed to identify the determinants of male partner involvement during antenatal care among pregnant women in Gedeo Zone, South Ethiopia.

**Methods:**

Community based unmatched case-control study was carried out from January to March 2019 among 804 (cases-402 and controls-402) selected pregnant women having antenatal follow up in Gedeo zone by stratified sampling technique. Data was collected using a pretested, structured, interviewer-administered questionnaire. A survey was conducted in the 22 selected kebeles in the Gedeo zone to identify cases and control. The data was entered using Epi-data and exported to SPSS (Statistical Package for Social Sciences) for analysis. Descriptive analysis like frequency, percentage, rates, and inferential analysis such as binary logistic regression are used. Statistical significance is declared at α < 0.05. The result is presented using text and tables.

**Results:**

Husband and maternal age difference (AOR = 1.12, 95% CI [1.06, 1.18]), maternal age at marriage (AOR = 0.86, 95% CI[0.81,0.93]), women empowerment (AOR = 0.20, 95% CI[0.13, 0.30]), type of nearby health facility (AOR = 4.94, 95% CI[1.67, 14.60]) and provider invitation of male partner to antenatal care examination room (AOR = 0.32, 95% CI[0.20, 0.51]) were determinants of male partner involvement during antenatal care.

**Conclusions:**

Age difference between husband and wife, age at marriage, women empowerment, type of nearby health facility and male invitation by health providers to antenatal care examination room determines male partner antenatal care involvement. Promoting women empowerment and inviting a male partner to antenatal care are recommended to encourage male involvement during antenatal care.

## Introduction

Maternal mortality is among the major concerns of the globe with special emphasis in developing countries. Every day in 2017, about 808 women died due to complications of pregnancy and childbirth. Almost all of these deaths occurred in low-resource settings, and most could have been prevented. The primary causes of death are hemorrhage, hypertension, infections, and indirect causes, mostly due to the interaction between pre-existing medical conditions and pregnancy [1]. To tackle these problems, different strategies are being implemented, including antenatal care (ANC).

Data from 2018 shows that there are only 83 countries all over the world where 75% of pregnant women had at least four antenatal care visits. The coverage in Africa ranges from lowest in South Sudan (17%) to highest in Ghana (87.3%) [2]. According to the recent 2019 Ethiopian mini demographic health survey only 43% of pregnant women had at least four ANC visits, while 74% of pregnant women received ANC only once [3].

Community and significant other’s support for pregnant mothers are vital to exploit all the available services under antenatal care [4]. Husband involvement during ANC is one of the supports expected from the husband or male partner which is composed of both health facility and household level involvement. In the household, males are involved in promoting a healthy lifestyle like avoiding un-prescribed drugs, alcohol drinking, smoking, and encouraging to take prescribed drugs, variety of food, having rest and visiting a health facility at the time of ANC appointment and in case of problems. At the facility level, the support starts from accompanying pregnant women to antenatal care clinic and attending the services. While attending ANC service, male partner is expected to actively participate on human immune deficiency virus (HIV) counseling and testing, health education session, birth preparedness and complication readiness plan and on related decision making with their wife [5,6].

The evidence concerning male involvement in antenatal care for their respective pregnant wives is meager across the world. According to different small-scale cross-sectional studies at different times and place male involvement ranges from 6% in Uganda to 86.8% in Rwanda [7,8]. Even in Ghana, a country with the highest antenatal care coverage the involvement of males, was limited to not more than 10% [9]. Whereas in Ethiopia it was not more than twenty percent according to studies from Harari and Mekelle focusing on the prevention of mother-to-child transmission (PMTCT) segment of ANC [10,11].

Male partner involvement had a different reputation, especially on maternal health service uptake. It improves the early commencement of antenatal care and provides a better chance to deliver ANC components [12,13]. Uptake of facility delivery and postnatal checkup also increase as a result of male involvement in ANC [14,15,16,17]. Male engagement in ANC minimizes the risk of postpartum depression [18].

According to available evidence, male involvement can be determined by different categories of factors such as socio-demographic, sociocultural, reproductive health and health facility-related [8,11,19,20]. But due to scanty of evidence, their level of influence was not known in much detail. Even most of the available studies look at male involvement merely concerning facility-level even only the PMTCT segment of antenatal care, which means they were not holistic. Whereas in this study male involvement during antenatal care will be measured by considering the level of involvement from household through facility level. Due to the above reason, this study will be important in identifying the determinant factors of male involvement during antenatal care.

## Methods

### Study Design and Area

A community based unmatched case-control study has been carried out from January to March 2019 in Gedeo, a Zone in the Southern Nations, Nationalities, and Peoples’ Region (SNNPR) of Ethiopia which is located at 6°07’38.2”N latitude and 38°16’37.8”E longitude. According to the 2009 population projection, this Zone has a total population of 1,112,951; with an area of 1,210.89 square kilometers and population density of 919.11. Only 10% are urban inhabitants and the rest live in the rural areas. Gedeo is among the top coffee and inset producing area in Ethiopia. It is a home of eight districts and 148 kebeles. Dilla town is the administrative center of Gedeo zone which is located 273kms south of Addis Ababa, the capital of Ethiopia. There are four public health hospitals in the Gedeo zone; one is a referral and teaching hospital while the other three are district hospitals. In total, there are 38 health centers and 146 health posts.

### Sample Size Determination

The sample size was calculated using Epi info statistical software considering: control to case ratio 1; Percent of control exposed (knowledge of their serostatus) 97.9%; Percent of cases exposed 93.4% from a study carried out in Mekelle, Northern Ethiopia; [10] 95% level of confidence; 80% power and 10% non-response. Finally, this generated, a total sample size of 804 (402 cases and 402 controls).

### Sampling Technique and Procedures

**Figure 1:**
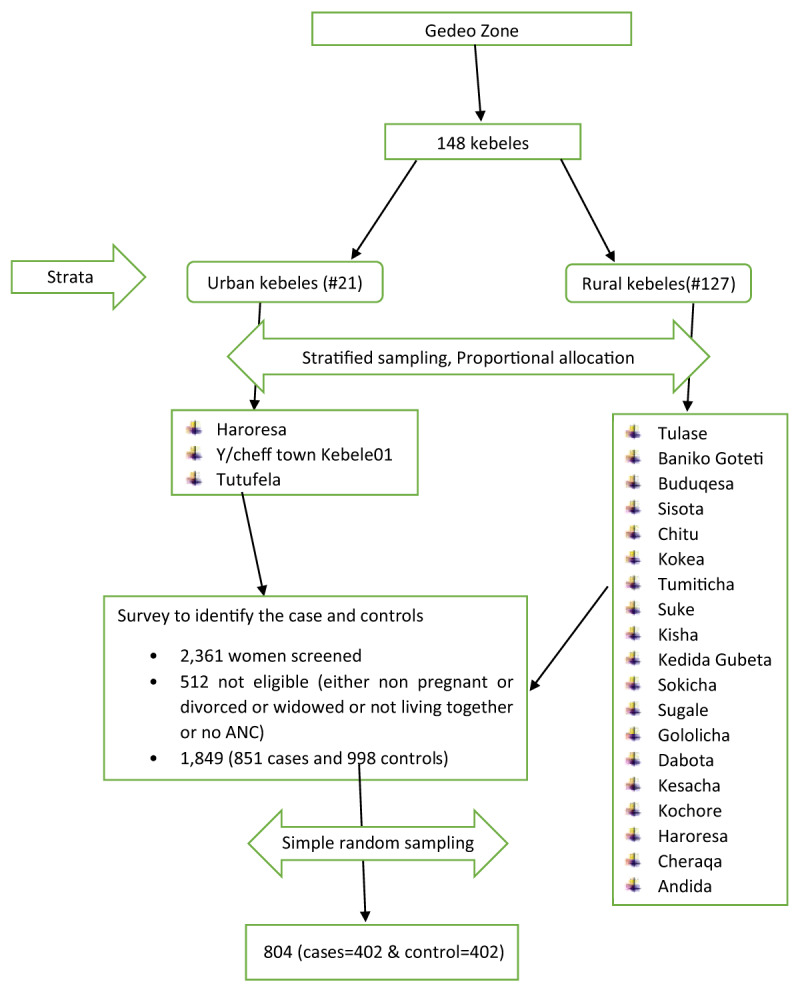
Schematic presentation of sampling procedure to study determinants of male partner involvement during antenatal care among pregnant women in Gedeo zone, South Ethiopia, 2019.

There are 148 kebeles (21 urban and 127 rural) in the Gedeo zone. Twenty-two kebeles were selected using a stratified sampling technique, considering urban and rural kebeles as strata. Then on each selected kebeles survey was carried out and a total of 2,361 women were screened. From this 1,849 (851 cases and 998 controls) were eligible for the study where the rest were dropped due to pregnancy status (196 non-pregnant), marital status (175 either divorced or widowed or not living together) and antenatal care status (141 had no antenatal care). Finally using simple random sampling technique individual pregnant women for both cases and controls were selected (***Figure 1***).

### Operational Definitions

#### Male partner involvement during antenatal care

It was composed of both health facility and household level involvement. A total of 14 items, nine for health facility level and five for household-level involvement was used. For each item, those who respond “Yes” were scored 1 and those who respond “No” scored 0. Male partner involvement was determined based on the mean score.

A male partner was considered as involved during antenatal care when the score was the mean score and above, while the score below the mean score was considered as not involved.

#### Cases

Pregnant women whose male partner was involved during their antenatal care based on the above criteria (score of mean value and above).

#### Controls

Pregnant women whose male partner was not involved during their antenatal care based on the above criteria (score below mean value).

#### Women’s empowerment

It was measured using three-dimension, namely economic, socio-familial and legal dimensions. A total of nine items, four for economic, three for socio-familial and two for legal dimension was used. The level of empowerment was determined based on the mean score; those women with a score of mean and above were categorized as empowered while those with a score of below mean score were considered as under empowered.

### Data Collection Procedures

Data was collected using a pretested, structured, interviewer-administered questionnaire. Initially, the instrument was designed in English, then translated to the local language and then back to English. The questionnaire was derived from related literature and has socio-demographic, social, reproductive health and health facility-related sections. A total of nine data collectors who completed at least high school were recruited. Two health professionals had supervised the whole data collection process. The data collection process had two-phases. In the first phase, a survey was carried out in the selected kebeles to identify the cases and controls using a set of structured questions prepared on male involvement. In the second phase, the actual data on socio-demographic, social and reproductive health were collected from randomly selected cases and controls.

### Data Processing and Analysis

The data was entered using Epi-data and exported to SPSS (Statistical Package for Social Sciences) software for analysis. Data were cleaned and explored to check accuracy and consistency and the necessary correction was made accordingly. Descriptive statistics; frequencies, percentages, mean and standard deviation were used to present the finding. To identify the determinant factors of male involvement, binary logistic regression was used. Variable with α < 0.25 on bivariate binary logistic regression and without multi-collinearity (r < 0.05) were candidates for multivariate binary logistic regression analysis. The model fitness was checked using Hosmer and Lemeshow test. Statistical significance was declared at α <0.05. The result was presented using text and tables.

## Results

### Socio-Demographic Characteristics

The mean age of the mothers was 26.9 ± 4.8 years, while 33.9 ± 6.4 years was the mean age of the male partner. Therefore, the mean age difference between the male partner and the mother was 6.9 ± 4.1 years. More cases (33.1%) than controls (6.5%) were urban residents. As per the maternal education, secondary school was attained more among controls (30.6%) which was triples of cases (10.2%) (***Table 1***).

**Table 1:** Socio-demographic characteristics of mothers and their male partner in Gedeo zone, South Ethiopia, 2019.


VARIABLES	CASE N (%)	CONTROL N (%)	TOTAL N (%)

Maternal Age			

<22	69(17.2)	53(13.2)	122(15.2)

22–26	117(29.1)	119(29.6)	236(29.4)

27–31	139(34.6)	188(46.8)	327(40.7)

32–36	54(13.4)	34(8.5)	88(10.9)

>36	23(5.7)	8(2)	31(3.9)

Residence			

Urban	133(33.1)	26(6.5)	159(19.8)

Rural	269(66.9)	376(93.5)	645(80.2)

Ethnicity			

Gedeo	278(69.2)	290(72.1)	568(70.6)

Amhara	41(10.2)	16(4)	57(7.1)

Oromo	44(10.9)	90(22.4)	134(16.7)

Other^#^	39(9.7)	6(1.5)	45(5.6)

Religion			

Orthodox	105(26.1)	33(8.2)	138(17.2)

Protestant	257(63.9)	352(87.6)	609(75.7)

Other^$^	40(10)	17(4.2)	57(7.1)

Maternal Education			

Can’t read & write	68(16.9)	78(19.4)	146(18.2)

Read & write	109(27.1)	99(24.6)	208(25.9)

Primary	42(10.4)	45(11.2)	87(10.8)

Secondary	41(10.2)	123(30.6)	164(20.4)

College & above	142(35.3)	57(14.2)	199(24.8)

Husband Education			

Can’t read & write	46(11.4)	44(10.9)	90(11.2)

Read & write	57(14.2)	29(7.2)	86(10.7)

Primary	47(11.7)	51(12.7)	98(12.2)

Secondary	64(15.9)	195(48.5)	259(32.2)

College & above	188(46.8)	83(20.6)	271(33.7)

Maternal Occupation			

Government Employee	141(35.1)	53(13.2)	194(24.1)

Merchant	120(29.9)	116(28.9)	236(29.4)

House wife	115(28.6)	210(52.2)	325(40.4)

Other^+^	26(6.5)	23(5.7)	49(6.1)

Husband Occupation			

Government Employee	201(50)	72(17.9)	273(34)

Merchant	88(21.9)	197(49)	285(35.4)

Farmer	82(20.4)	56(13.9)	138(17.2)

Daily labor	31(7.7)	77(19.2)	108(13.4)

Wife order			

First	309(76.9)	336(83.6)	645(80.2)

Above first	93(23.1)	66(16.4)	159(19.8)

Wealth index			

Poorest	124(30.8)	37(9.2)	161(20)

Poor	115(28.6)	52(12.9)	167(20.8)

Medium	93(23.1)	61(15.2)	154(19.2)

Rich	56(13.9)	92(22.9)	148(18.4)

Richest	14(3.5)	160(39.8)	147(21.6)


#: Includes Gurage, Sidama, Wolayta & Tigre; $: Includes Muslim & no religion; +: Includes farmers, home maid & daily laborer.

### Women Empowerment

In general, more than half (57.2%) of the mothers were empowered, in which more cases (80.8%) than controls (33.6%) were empowered. The majority of controls (80.6%) than cases (36.1%) were legally under empowered (***Table 2***).

**Table 2:** Women empowerment of mothers in Gedeo zone, South Ethiopia, 2019.


VARIABLES	CASE N (%)	CONTROL N (%)	TOTAL N (%)

Women economic empowerment	Mean score– 2.8 ± 1.1		

Under empowered	60(14.9)	209(52)	269(33.5)

Empowered	342(85.1)	193(48)	535(66.5)

Women social empowerment	Mean score– 1.8 ± 0.9		

Under empowered	116(28.9)	143(35.6)	259(32.2)

Empowered	286(71.1)	259(64.4)	545(67.8)

Women legal empowerment	Mean score– 1.1 ± 0.9		

Under empowered	145(36.1)	324(80.6)	469(58.3)

Empowered	257(63.9)	78(19.4)	335(41.7)

Overall women empowerment	Mean score– 5.7 ± 2		

Under empowered	77(19.2)	267(66.4)	344(42.8)

Empowered	325(80.8)	135(33.6)	460(57.2)


### Reproductive Health and Health Facility Characteristics

The mean age of the mothers at marriage was 20.3 ± 2.8 year, where their mean marital length of stay was 7.6 ± 5 years. The average number of pregnancies experienced by the mothers was 3 ± 2.2, while their mean age at first birth was 21.3 ± 2.6 years. The mean order of current pregnancy of the mothers was 2.9 ± 1.5, while averagely at 3.6 ± 1.8 months of the stage they initiated antenatal care follow up. The mean estimated time to reach the nearest health facility for mothers on their bare feet was 23.6 ± 16 minutes. The estimated average waiting time at the antenatal care clinic was 35.3 ± 33.4 minutes while on average there were 3.1 ± 3.7 pregnant mothers for each mother to wait before getting care at ANC clinic (***Table 3***).

**Table 3:** Reproductive health and health facility characteristics of mothers in Gedeo zone, South Ethiopia, 2019.


VARIABLES	CASE N (%)	CONTROL N (%)	TOTAL N (%)

ANC follow up for previous pregnancies	360	308	668

Yes	311(86.4)	287(93.2)	598(89.5)

No	49(13.6)	21(6.8)	70(10.5)

Male partner accompany to ANC clinic for previous pregnancies	358	308	666

Yes	125(34.9)	101(32.8)	226(33.9)

No	233(65.1)	207(67.2)	440(66.1)

Birth place for previous pregnancies	360	308	668

Home	197(54.7)	214(69.5)	411(61.5)

Health center	117(32.5)	51(16.6)	168(25.1)

Hospital	28(7.8)	8(2.6)	36(5.4)

Other	18(5)	35(11.4)	53(7.9)

Current pregnancy intension			

Planned	286(71.1)	337(83.8)	623(77.5)

Unplanned	116(28.9)	65(16.2)	181(22.5)

Male partner invitation into ANC clinic exam room			

Yes	115(28.6)	51(12.7)	166(20.6)

No	287(71.4)	351(87.3)	638(79.4)

Nearby health facility			

Hospital	76(18.9)	30(7.5)	106(13.2)

Health center	248(61.7)	173(43)	421(52.4)

Health post	54(13.4)	191(47.5)	245(30.5)

Private clinic	24(6)	8(2)	32(4)

Availability of waiting area in the ANC clinic			

Yes	373(92.8)	388(96.5)	761(94.7)

No	29(7.2)	14(3.5)	43(5.3)


### Determinates of Male Involvement During Antenatal Care

The husband and maternal age difference, maternal occupation, the order of mother among husband’s wives, women empowerment, maternal age at marriage, gravida, current antenatal care initiation time, type of nearby health facility, estimated time to reach nearby health facility and invitation of the male partner to antenatal care examination room to attend by the health professionals were variables candidate for multivariate binary logistic regression analysis after passing bivariate binary logistic regression (α < 0.25) and multi-collinearity (r < 0.5) analysis. From these variables husband and maternal age difference, maternal age at marriage, women empowerment, type of nearby health facility and male partner invitation to ANC examination room by healthcare providers maintained their statistical significance in multivariate binary logistic regression and considered as determinants of male partner involvement during antenatal care.

A unite increase in age difference between husband and wife increases the odds of male partner involvement at antenatal care by 12% (AOR = 1.12, 95% CI [1.06, 1.18]). Whereas a unite increase in maternal age at marriage reduced the odds of male partner involvement during antenatal care by 14% (AOR = 0.86, 95% CI [0.81, 0.93]). Husbands of under empowered mothers were 80% less likely hood to be involved during antenatal care of their wife as compared to husbands of empowered ones (AOR = 0.20, 95% CI[0.13, 0.30]). The type of nearby health facility for the mother is another determinant factor for male partner involvement during antenatal care. Those mothers with nearby health facilities being private clinics were found five times more likely for their male partner to be involved during their antenatal care as compared to those with nearby public hospitals (AOR = 4.94, 95% CI[1.67, 14.60]). Whereas mothers with nearby health facilities being health posts were 70% less likely for their male partner to be involved during their antenatal care than those with nearby public hospitals (AOR = 0.30, 95% CI[0.16, 0.58]). The absence of male partner invitation to ANC clinic examination room by the healthcare provider has reduced the odds of male partner involvement during their wife antenatal care by 68% (AOR = 0.32, 95% CI [0.20, 0.51]) (***Table 4***).

**Table 4:** Determinants of male partner involvement during antenatal care among pregnant women in Gedeo zone, South Ethiopia, 2019.


VARIABLES	CASE N (%)	CONTROL N (%)	COR (95% CI)	AOR (95% CI)

Age difference			1.2(1.15, 1.26)**	1.12(1.06, 1.18)**

Maternal Occupation				

Government Employee	141(35.1)	53(13.2)	1	1

Merchant	120(29.9)	116(28.9)	0.39(0.26, 0.58)**	0.92(0.55, 1.53)

House wife	115(28.6)	210(52.2)	0.21(0.14, 0.30)**	0.80(0.49, 1.33)

Other^+^	26(6.5)	23(5.7)	0.43(0.22, 0.81)*	1.09(0.46, 2.57)

Overall women empowerment				

Under empowered	77(19.2)	267(66.4)	0.12(0.09, 0.17)**	0.20(0.13, 0.30)**

Empowered	325(80.8)	135(33.6)	1	1

Wife order				

First	309(76.9)	336(83.6)	1	1

Above first	93(23.1)	66(16.4)	1.53(1.08, 2.18)*	1.04(0.66, 1.63)

Maternal age at marriage			0.82(0.77, 0.87)**	0.86(0.81, 0.93)**

Gravida			1.21(1.11, 1.33)**	1.07(0.96, 1.19)

Current pregnancy intension				

Planned	286(71.1)	337(83.8)	1	1

Unplanned	116(28.9)	65(16.2)	2.10(1.49, 2.96)**	1.48(0.94, 2.32)

ANC commencement stage			1.12(1.03, 1.21)*	1.09(0.97, 1.22)

Nearby health facility				

Hospital	76(18.9)	30(7.5)	1	1

Health center	248(61.7)	173(43)	0.57(0.36, 0.90)*	1.13(0.64, 1.98)

Health post	54(13.4)	191(47.5)	0.11(0.07, 0.19)**	0.30(0.16, 0.58)**

Private clinic	24(6)	8(2)	1.18(0.48, 2.93)	4.94(1.67, 14.60)*

Time to reach nearby health facility			1.02(1.01, 1.03)**	1.00(0.99, 1.01)

Male partner invitation into ANC clinic exam room				

Yes	115(28.6)	51(12.7)	1	1

No	287(71.4)	351(87.3)	0.36(0.25, 0.52)**	0.32(0.20, 0.51)**


*:α < 0.05; **:< 0.001; +: Includes farmers, home maid & daily laborer.

## Discussion

In this study, the age difference between husband and mother, maternal age at marriage, women empowerment, type of nearby health facility and male partner invitation by the healthcare providers to antenatal care examination room were determinants of male partner involvement during antenatal care.

According to this study, an increase in age difference between husband and wife was found to increase the odds of male partner involvement. This may be related to the issue of caring and the need for support. As the age of a male partner is significantly higher than his wife, he may think that his wife needs to be cared and supported during her pregnancy. In support of this, another finding of this study indicated that an increase in maternal age at marriage was associated with the reduction of male involvement. It may be due to that paternal concern for family health may reduce as the maternal age increase considering as they are capable of it by themselves. In contrast, a qualitative study from Gambia indicated as a large age difference hinder male partner involvement [21]. The difference may be due to study design and socio-cultural differences.

Maternal level of empowerment was another pertinent determinant of male partner involvement during antenatal care. As per the finding of this study under-empowerment of mothers was reduced male partner involvement during antenatal care by 80%. It is aligned with finding from Uganda and Burkina Faso since there was a positive association between empowered women and male involvement [7]. This might be due to, as the mother’s level of decision-making participation or contribution for life is minimized, their ability to negotiate their male partner to involve during their antenatal care may reduce. Even those under empowered women may have antenatal care hidden from their respective male partners due to fear of restriction from health care. On contrary, in Malawi empowered women were less likely for their male partners to be involved during their respective antenatal care [7]. The difference may be due to socio-cultural discrepancy.

The type of nearby health facility for the mother determines male partner involvement during antenatal care. When a private clinic was the nearby health facility there were five times more likely hood of male partner involvement during antenatal care as compared to a public hospital. This may be related to crowdedness, since public facilities were considered more crowded and may not be convenient for the pre-occupied male partner to involve during their respective wife antenatal follow up. In contrast, the odds of male partner involvement during antenatal care were reduced by 70% for nearby health post than a public hospital. This may be related to the perceived level of quality of care and the welcoming of the health providers at the health post. But as far as the knowledge of the authors there was no evidence on this variable.

The absence of male partner invitation to the antenatal care examination room by the healthcare provider was found to reduce the odds of male partner involvement during antenatal care by 68%. This may be because, unless partners accompanied with their pregnant wives to the antenatal clinic are are actively participated with the care like counseling provided to their wives, simple accompaniment is meaningless. It may discourage them and think as they’re escorting their wives weren’t accepted by the health professionals [9,22]. It was supported by a qualitative study from Guinea in which friendly health workers were indicated as they were supportive of male partner involvement during antenatal care [23]. Similarly, a systematic review from English revealed that male unfriendly PMTC services were among the barriers to male partner involvement [24].

Solely considering women only as a study participant by ignoring the male partner was the limitation of the study. Also, most of the variables were measured based on the subjective response of the women and the ability to remind things which may introduce recall bias.

## Conclusions

The age difference between husband and wife, age at marriage, women empowerment, type of nearby health facility and male invitation by healthcare providers to antenatal care examination room determines male partner antenatal care involvement for their respective pregnant wives. Promoting women empowerment and inviting a male partner to ANC are recommended to encourage male involvement during antenatal care.

## Data Accessibility Statement

We have sent all the available data and we do not want to share the raw data as we are conducting a related study.

## Abbreviations

ANC: Antenatal Care

AOR: Adjusted Odds Ratio

CI: Confidence Interval

COR: Crude Odds Ratio

HIV: Human Immune Deficiency Virus

PMTCT: Prevention of Mother to Child Transmission

SPSS: Statistical Package for Social Sciences
